# Association of sleep quality with cognitive dysfunction in middle-aged and elderly adults: a cross-sectional study in China

**DOI:** 10.3389/fnagi.2024.1417349

**Published:** 2024-09-16

**Authors:** Mengyang Jiang, Yang Liu, Xin Wang, Yuhe Liu, Xuan Deng, Xiaoyu Zhang, Baoguo Wang

**Affiliations:** ^1^Department of Anesthesiology, Sanbo Brain Hospital, Capital Medical University, Beijing, China; ^2^Department of Thoracic Surgery, Air Force Medical Center, PLA, Beijing, China; ^3^Department of Anesthesiology, Peking University Cancer Hospital & Institute, Beijing, China; ^4^Department of Biochemistry and Molecular Biology, Hengyang Medical School, University of South China, Hengyang, China; ^5^Clinical Research Institute, Shanghai General Hospital, Shanghai Jiao Tong University School of Medicine, Shanghai, China

**Keywords:** sleep quality, cognitive dysfunction, sleep duration, staying up, sleep latency

## Abstract

**Objectives:**

Sleep is an indispensable part of human health, which can help us to restore physical strength, enhance immunity and maintain nervous system stability. The relationship between sleep quality and cognitive dysfunction is unclear, especially at the community population level. This study aims to explore the association between sleep quality and cognitive dysfunction.

**Methods:**

A total of 5,224 community residents were enrolled in this cross-sectional study. Cognitive function was assessed by the Mini-Mental State Examination (MMSE). Sleep quality was assessed by the multidimensional sleep questionnaire. Multivariate logistic regression was used to analyze the association between sleep quality and cognitive dysfunction. The adjusted models took into account relevant demographic, clinical, and sleep variables.

**Results:**

A total of 3,106 participants were enrolled in this study, of whom 463 (15%) had cognitive dysfunction. Total sleep duration, staying up, sleep latency, number of awakenings, and history of sleep medications were associated with cognitive dysfunction in unadjusted models, and these effects were consistent after adjustment. First, those who slept 6–7.9 h per day (OR = 0.57, 95% CI 0.40 to 0.80, *p* = 0.001) had a lower risk for cognitive dysfunction compared to those who slept less than 6 h per day. Second, participants who stayed up more than 10 times over the 3 months (OR = 1.90, 95% CI 1.20 to 3.00, *p* = 0.006) were more likely to suffer cognitive dysfunction than those who never stayed up. Third, we also found that participants with sleep latencies of 16–30 min were less likely to experience cognitive dysfunction than those with sleep latencies of less than 16 min after adjusting confounders (OR = 0.33, 95% CI 0.23 to 0.47, *p* < 0.001). Fourth, participants who woke up once (OR = 1.65, 95% CI 1.19 to 2.30, *p* = 0.003) and three or more times (OR = 2.34, 95% CI 1.25 to 4.36, *p* = 0.008) after falling asleep had a higher risk than those who did not wake up at night. Last, participants taking sleep medication (OR = 2.97, 95% CI 1.19 to 7.45, *p* = 0.020) were more vulnerable to cognitive dysfunction, relative to participants without taking any medications.

**Conclusion:**

Our results suggest that after adjustment for potential confounding variables, poor sleep quality is associated with cognitive dysfunction.

## Introduction

Cognitive dysfunction is defined as a decline in cognitive domains such as memory, executive function, attention, language, and visual spatial function (Millan et al., 2012). Cognitive dysfunction can be caused by a variety of factors, such as brain damage, neurological disorders, side effects of medications, etc. (El Husseini et al., 2023; Zhang et al., 2023). Clinically, cognitive dysfunction may be manifested as memory loss, inattention, slow thinking, language disorders and other symptoms (Perez-Carbonell and Iranzo, 2023). The rate of conversion from cognitive dysfunction to dementia were much higher than the normal cognitive population, which has become a burden on society (Song et al., 2020). For these reasons, early intervention for modifiable risk factors is becoming increasingly important.

Sleep is an indispensable part of human health, which can help us restore physical strength, enhance immunity, and maintain nervous system stability (Liu et al., 2020; Sejbuk et al., 2022; Mahon and Glennon, 2023). Previous studies have found that sleep is involved in cognitive processes and alterations in sleep may lead to clinical cognitive impairment (Liu et al., 2020). Sleep is a complex physiological state that is accompanied by changes in neural network activity and the neurochemical environment (Simon et al., 2022). A systematic review of 71 studies showed that sleep disorders, including insomnia, sleep fragmentation and obstructive sleep apnea syndrome, could result in cognitive dysfunction (Casagrande et al., 2022). However, the relationship between sleep quality and cognitive dysfunction is inconsistent. A cohort study conducted in 1,629 adults between 48 and 91 years of age found that the presence of sleep disturbance did not significantly increase the risk of diagnostic conversion in cognition normal, early mild cognitive impairment, or late mild cognitive impairment participants, which means sleep disturbance could not predict subsequent cognitive decline (Mecca et al., 2018). Therefore, the relationship between the sleep quality and cognitive dysfunction needs to be further explored with a larger sample size study.

A survey study by the China Sleep Research Association shows that the prevalence of sleep disorders among adults in China is as high as 38.2%, and more than 300 million people suffer from sleep problems (Zhou et al., 2023). Sleep disorders and cognitive dysfunction are common in China. Long-term sleep disorders can easily induce neuroinflammation, which is one of the most important mechanisms of impaired cognitive function. We hypothesize that people with poor sleep quality are more likely to suffer from cognitive dysfunction. Therefore, we performed a cross-sectional study to explore the relationship between sleep quality and cognitive dysfunction in the Chinese population. Our findings will provide clinicians with direct evidence to reduce the occurrence of cognitive dysfunction and the socio-economic burden associated with cognitive dysfunction.

## Methods

### Study design

This study was a community-based cross-sectional study focusing on the sleep quality and cognitive dysfunction. The data were derived from a Jidong cognitive impairment cohort study which were collected by us in 2019. Detailed information about the cohort study has been published (Song et al., 2020). This study was performed in accordance with the guidelines described by the Helsinki Declaration and was approved by the Ethics Committees of Kailuan General Hospital of Tangshan City, the Staff Hospital of Jidong Oil-field Branch, China National Petroleum Corporation (No. 2013 YILUNZI 1).

### Participants

Participants received a health examination and clinical interview in the Staff Hospital, Jidong Oilfield Branch, China National Petroleum Corporation. All participants aged more than or equal to 40 years will be included in the study. Participants were excluded if they lack information of educational level, sleep assessment and cognitive functioning assessment questionnaires.

### Measurement of sleep quality

In this study, we evaluated the quality of the participant’s sleep through five parameters that can reflect the structure and duration of sleep in a dimensional way. The five sleep parameters were total sleep duration, frequency of staying up, sleep latency, number of awakenings after sleep onset, and history of sleep medication, investigated by face-to-face interviews. Total sleep duration was assessed by the question, ‘What is the average duration of your sleep per day in the last 3 months? (<6 h, 6–7.9 h, ≥8 h)’. Frequency of staying up was assessed by the question, ‘How many times have you stayed up in the last 3 months? (0, 1–5, 6–10, ≥11)’. Sleep latency was assessed by the question, ‘How long do you usually take to fall asleep? (0-15 min, 16-30 min, 31-60 min, >60 min)’. Number of awakenings after sleep onset was assessed by the question, ‘How many times do you wake up during your sleep? (0, 1, 2, ≥3)’. History of sleep medication was assessed by the question, ‘Have you ever taken any sleep medications? (Yes, no)’.

### Measurement of cognitive dysfunction

Assessment of cognitive function was performed using the Chinese version of Mini-Mental State Examination (MMSE) (Li et al., 2016). The MMSE is the most common used cognitive screening tool in the world and is able to assess five dimensions including orientation, memory, language, recall, attention and computation. Its total score was 30, with higher scores indicating better cognitive function. The widely accepted cut-offs of MMSE score for cognitive dysfunction were defined based on educational level: <18 for illiteracy; <21 for primary school graduates; <25 for junior school graduates or above.

### Covariates

The demographic characteristics, including age, sex, body mass index, educational level, alcohol drinking, smoking, and exercise habits were obtained from the self-reported questionnaire. The required relevant medical history and laboratory data were obtained from the medical chart.

### Statistical analyses

Categorical variables were described as percentage (%) and compared by Chi-square tests. Normal distributed continuous variables were described as the mean ± standard deviation (SD) and compared with ANOVA or t test. Multivariate logistic regression models were used to calculate the odds ratio (OR) and 95% confidence interval (95% CI) for cognitive dysfunction. We conducted univariate analyses of the relationship between all variables and cognitive dysfunction. Statistically differentiated variables in the univariate analysis were subsequently included in multivariate logistic regression models. Following the recommendations provided by Reporting on Observational Studies in Strengthening Epidemiology, three weighted multivariate logistic regression models were created to acquire a more comprehensive understanding of the association between sleep quality and cognitive dysfunction. Crude model was not adjusted for any confounders, Model 1 was adjusted for age and sex, and Model 2 was adjusted for all confounders, including age, sex, BMI, hypertension, diabetes, hyperlipidemia, drink, smoke, glucose, NLR, exercise. Stratified analyses were used to estimate the association between sleep quality and cognitive function, stratified by age, sex, BMI, hypertension, diabetes, hyperlipidemia, and smoke. Statistical analyses were performed using SAS software (Version 9.4, SAS Institute, Cary, NC, United States). All statistical tests were 2-sided, and significance levels were 0.05.

## Results

### General and sleep characteristics of participants

**Figure 1:**
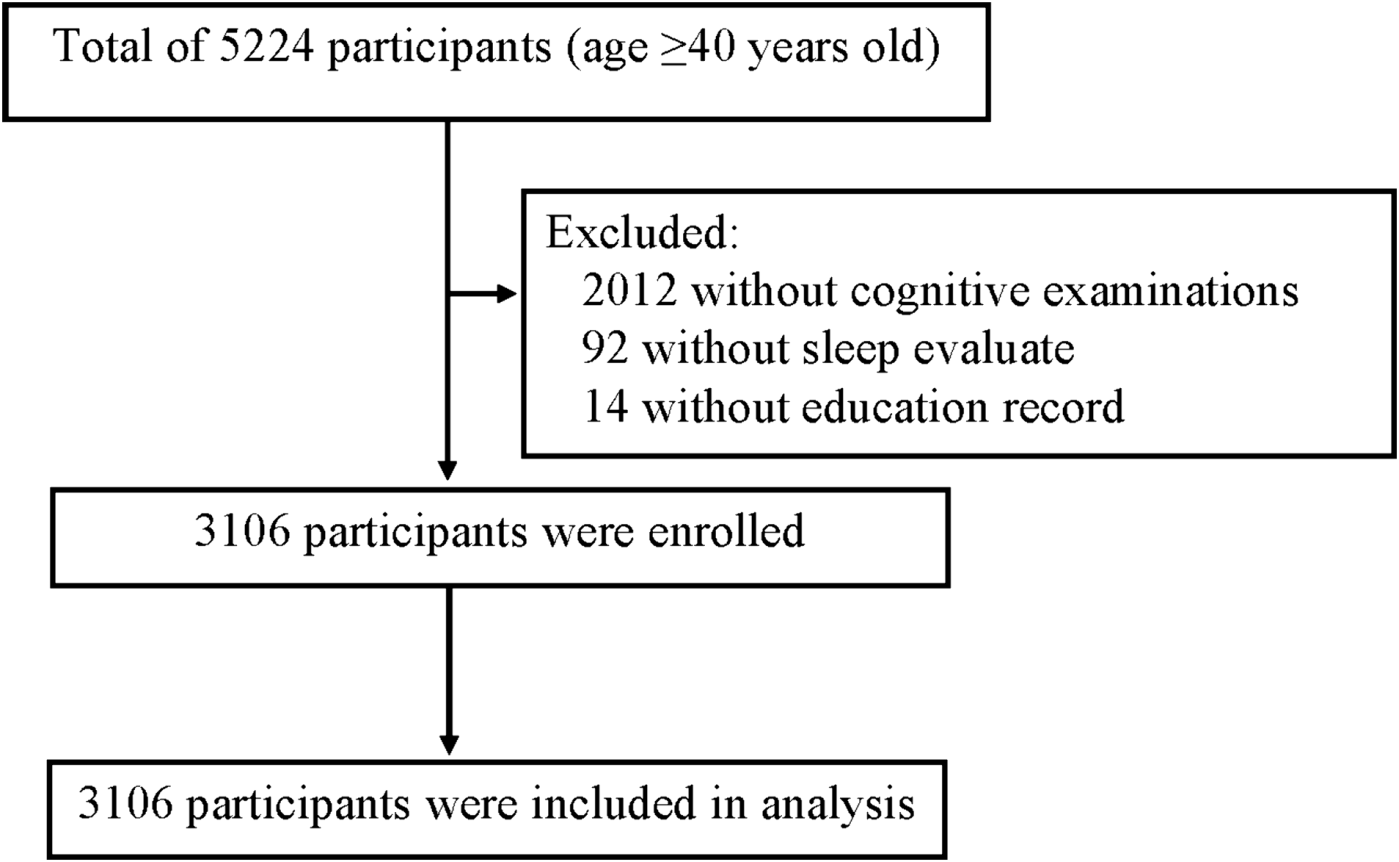
The flowchart of the study.

Of 5,224 participants underwent health examination and clinical interview, 3,106 participants were recruited (Figure 1). The general characteristics of participants are shown in Table 1. Among all these participants, the majority were between 40 and 59 years old, and 48.9% of them were male. According to the assessment criteria of MMSE, 463 (15%) participants had cognitive dysfunction. The results of univariate analyses shown that age (*p* < 0.001), BMI (*p* = 0.001), hypertension (*p* < 0.001), diabetes (*p* = 0.024), hyperlipidemia (*p* = 0.009), glucose (*p* = 0.026), and exercise habit (*p* < 0.001) were associated with cognitive dysfunction.

**Table 1:** Baseline characteristics of participants according to cognitive dysfunction.

Characteristics	Total	Cognitive dysfunction		*p* value
		No	Yes	
*N*, %	3,106 (100)	2,643 (85)	463 (15)	
Age (years)				<0.001
40–59	2058 (66.3)	1864 (70.5)	194 (41.9)	
60–69	835 (26.9)	644 (24.4)	191 (41.3)	
≥70	213 (6.9)	135 (5.1)	78 (16.9)	
Sex				0.396
Male (*n*, %)	1,520 (48.9)	1,285 (48.6)	235 (50.8)	
Female (*n*, %)	1,586 (51.1)	1,358 (51.4)	228 (49.2)	
BMI (kg/m^2^)				0.001
<24	1,272 (41.0)	1,114 (42.2)	158 (34.1)	
≥24	1834 (59.1)	1,529 (57.9)	305 (65.9)	
Education level (*n*, %)				0.131
Illiteracy/primary	41 (1.3)	38 (1.4)	3 (0.7)	
Middle school	169 (5.4)	137 (5.2)	32 (6.9)	
College or above	2,896 (93.2)	2,468 (93.4)	428 (92.4)	
Hypertension (*n*, %)	1,293 (41.6)	1,046 (39.6)	247 (53.4)	<0.001
Diabetes (*n*, %)	445 (14.3)	363 (13.7)	82 (17.7)	0.024
Hyperlipidemia (*n*, %)	1733 (55.8)	1,449 (54.8)	284 (61.3)	0.009
Drinking (*n*, %)	536 (17.6)	455 (17.6)	81 (17.7)	0.943
Smoking (*n*, %)	632 (20.4)	536 (20.3)	96 (20.7)	0.823
Glucose (mmol/L)	5.8 ± 1.6	5.8 ± 1.5	6.0 ± 1.7	0.026
Hemoglobin (g/L)	145.2 ± 16.1	145.1 ± 16.4	146.1 ± 14.3	0.215
Neutrophil/lymphocyte ratio	1.9 ± 0.7	1.9 ± 0.8	1.8 ± 0.7	0.264
Exercise habit (*n*, %)				<0.001
<1 peer week	600 (24.0)	572 (25.5)	28 (11.0)	
1–2 peer week	280 (11.2)	269 (12.0)	11 (4.3)	
3–4 per week	218 (8.7)	198 (8.8)	20 (7.8)	
>4 peer week	1,402 (56.1)	1,206 (53.7)	196 (76.9)	

BMI: Body mass index.

The sleep quality of participants is shown in Table 2. The results of univariate analyses shown that total sleep duration (*p* < 0.001), sleep latency (*p* < 0.001), number of awakenings after sleep onset (*p* < 0.001), and history of sleep medication (*p* < 0.001) were associated with cognitive dysfunction.

**Table 2:** Sleep quality of participants according to cognitive dysfunction.

Characteristics	Total	Cognitive dysfunction		*P* value
		No	Yes	
*N*, %	3,106 (100)	2,643 (85)	463 (15)	
Total sleep duration				<0.001
<6 h	518 (16.7)	400 (15.1)	118 (25.5)	
6–7.9 h	2,308 (74.3)	1997 (75.6)	311 (67.2)	
>8 h	280 (9.0)	246 (9.3)	34 (7.3)	
Sleep latency				<0.001
0-15 min	1,692 (54.5)	1,355 (51.3)	337 (72.8)	
16-30 min	1,168 (37.6)	1,093 (41.4)	75 (16.2)	
30-60 min	158 (5.1)	137 (5.2)	21 (4.5)	
>1 h	88 (2.8)	58 (2.2)	30 (6.5)	
Number of awakenings				<0.001
0	1,304 (42.0)	1,137 (43.0)	167 (36.1)	
1	1,401 (45.1)	1,190 (45.0)	211 (45.6)	
2	277 (8.9)	223 (8.4)	54 (11.7)	
≥3	124 (4.0)	93 (3.5)	31 (6.7)	
Frequency of staying up				0.466
0	2,558 (82.4)	2,172 (82.2)	386 (83.4)	
1–5	199 (6.4)	176 (6.7)	23 (5.0)	
6–10	58 (1.9)	51 (1.9)	7 (1.5)	
≥11	29 (9.4)	244 (9.2)	47 (10.2)	
Sleep medication				<0.001
No	3,074 (99.0)	2,623 (99.2)	451 (97.4)	
Yes	32 (1.0)	20 (0.8)	12 (2.6)	

### Associations between sleep quality and cognitive dysfunction

Logistic regression analyses were further performed to assess the associations between five variables related to sleep quality and cognitive dysfunction (Table 3). First, those who slept 6–7.9 h per day (OR = 0.57, 95% CI 0.40 to 0.80, *p* = 0.001) had a lower risk for cognitive dysfunction compared to those who slept less than 6 h per day. Second, we also found that participants with sleep latencies of 16–30 min were less likely to experience cognitive dysfunction than those with sleep latencies of less than 16 min in model 2 after adjusting confounders (OR = 0.33, 95% CI 0.23 to 0.47, *p* < 0.001). Third, participants who woke up once (OR = 1.65, 95% CI 1.19 to 2.30, *p* = 0.003) and three or more times (OR = 2.34, 95% CI 1.25 to 4.36, *p* = 0.008) after falling asleep had a higher risk of cognitive dysfunction than those who did not wake up at night. Fourth, participants who stayed up more than 10 times over the 3 months (OR = 1.90, 95% CI 1.20 to 3.00, *p* = 0.006) were more likely to suffer cognitive dysfunction than those who never stayed up. Last, the association between history of sleep medications and cognitive dysfunction was steady in three models. Participants taking sleep medication (OR = 2.97, 95% CI 1.19 to 7.45, *p* = 0.020) were more vulnerable to cognitive dysfunction, relative to participants without taking any medications.

**Table 3:** Association between sleep quality and cognitive dysfunction.

	Crude model		Model 1		Model 2	
	Unadjusted OR (95% CI)	*P*	Adjusted OR (95% CI)	*P*	Adjusted OR (95% CI)	*P*
Total sleep duration (<6 h)	Ref.		Ref.		Ref.	
Total sleep duration (6–7.9 h)	0.53(0.42–0.67)	<0.001	0.68 (0.53–0.88)	0.003	0.57 (0.40–0.80)	**0.001**
Total sleep duration (>8 h)	0.47 (0.31–0.71)	<0.001	0.66 (0.39–0.92)	0.019	0.58 (0.33–1.00)	0.052
P for trend		<0.001		0.003		**0.007**
Sleep latency (0-15 min)	Ref.		Ref.		Ref.	
Sleep latency (16-30 min)	0.28 (0.21–0.36)	<0.001	0.33 (0.25–0.43)	<0.001	0.33 (0.23–0.47)	**<0.001**
Sleep latency (30-60 min)	0.62 (0.38–0.99)	0.046	0.63 (0.38–1.02)	0.059	0.53 (0.27–1.06)	0.074
Sleep latency (>1 h)	2.08 (1.32–3.28)	0.002	1.69 (1.05–2.72)	0.032	1.30 (0.62–2.72)	0.490
P for trend		<0.001		0.002		**0.001**
Number of awakenings (0)	Ref.		Ref.		Ref.	
Number of awakenings (1)	1.21 (0.97–1.50)	0.091	1.07 (0.85–1.34)	0.578	1.65 (1.19–2.30)	**0.003**
Number of awakenings (2)	1.65 (1.18–2.13)	0.004	1.27 (0.89–1.80)	0.184	1.64 (0.98–2.74)	0.060
Number of awakenings (≥3)	2.27 (1.47–3.52)	<0.001	1.52 (0.96–2.40)	0.074	2.34 (1.25–4.36)	**0.008**
P for trend		<0.001		0.052		**0.001**
Frequency of staying up (None)	Ref.		Ref.		Ref.	
Frequency of staying up (1–5)	0.74 (0.47–1.15)	0.179	1.09 (0.69–1.74)	0.709	0.79 (0.37–1.67)	0.530
Frequency of staying up (6–10)	0.77 (0.35–1.72)	0.526	1.29 (0.56–2.97)	0.550	0.71 (0.17–3.11)	0.656
Frequency of staying up (≥11)	1.08 (0.78–1.51)	0.633	1.69 (1.19–2.41)	0.004	1.90 (1.20–3.00)	**0.006**
P for trend		0.960		0.004		**0.016**
Sleep medication						
No	Ref.		Ref.		Ref.	
Yes	3.49 (1.69–7.19)	<0.001	2.70 (1.26–5.78)	0.011	2.97 (1.19–7.45)	**0.020**
P for trend		<0.001		0.011		**0.020**

Bold values indicate statistically significant in the model 2.

### Stratified analysis

First, we performed stratified studies on other risk factors for cognitive impairment based on the total sleep duration (Supplementary Table S1). Stratified analysis found that there was an interaction between age and total sleep duration of 6–7.9 h (*p* = 0.042). In the subgroup of total sleep duration of 6–7.9 h, age between 40–59 and 60–69 were protective factors for cognitive dysfunction (OR = 0.44, 95% CI 0.25 to 0.78, *p* = 0.005; OR = 0.47, 95% CI 0.29 to 0.76, *p* = 0.002). Meanwhile, there was an interaction between BMI and total sleep duration greater than 8 h (*p* = 0.043). In the subgroup of total sleep duration greater than 8 h, BMI greater than or equal to 24 were protective factors for cognitive dysfunction (OR = 0.39, 95% CI 0.19 to 0.79, *p* = 0.010).

Second, stratified analysis found that there was an interaction between age and sleep latency greater than 1 h (*p* = 0.031) (Supplementary Table S2). In the subgroup of sleep latency greater than 1 h, age between 40 and 59 was a risk factor for cognitive dysfunction (OR = 4.02, 95% CI 1.17 to 13.84, *p* = 0.028).

Third, stratified analysis found that there was an interaction between age and the waking up once in the night (*p* < 0.001) (Supplementary Table S3). In the subgroup of number of waking up once in the night, age between 40–59 and greater than 70 were risk factors for cognitive dysfunction (OR = 3.14, 95% CI 1.84 to 5.35, *p* < 0.001; OR = 4.14, 95% CI 1.33 to 12.88, *p* = 0.014). Meanwhile, there was an interaction between smoking and the waking up once in the night (*p* = 0.005). In the subgroup of waking up once in the night, no smoking history was a risk factor for cognitive dysfunction (OR = 2.19, 95% CI 1.48 to 3.25, *p* < 0.001).

## Discussion

In this community-based cross-sectional study in China, we found that sleep quality was associated with cognitive dysfunction. Participants with 6–7.9 h total sleep duration and 16–30 min sleep latency had lower risk of suffering cognitive dysfunction. Besides, participants who were awake at night more frequently, stayed up late more often, and had a history of taking sleep medication had a higher incidence of cognitive dysfunction.

In China, the incidence of sleep disorders is close to 40 percent, and the trend is increasing year by year, which has become a common health problem (Lu et al., 2019). Sleep disorders mainly include insomnia, sleep apnea syndrome, sleep rhythm disorder and other types (Pavlova and Latreille, 2019). Sleep disorders can cause serious harm to both physical and mental health. Long-term sleep problems can lead to the dysfunction of various body systems and increase the risk of chronic diseases such as cardiovascular disease, diabetes, and obesity (Javaheri and Redline, 2017; Schipper et al., 2021). In addition, lack of sleep can also reduce the function of the immune system and increase the likelihood of contracting diseases. On the psychological side, sleep disorders can lead to mood swings, anxiety, depression, and other psychological problems, and even affect memory and cognitive function (Chance Nicholson and Pfeiffer, 2021). Currently, the role of sleep problems in cognitive function has drawn increasing attention. Previous studies have confirmed that cognitive dysfunction may be the early manifestation of some nervous system diseases, such as Alzheimer’s disease and cerebrovascular disease (Iadecola and Gottesman, 2019). These diseases can lead to a gradual decline in cognitive function, seriously affect the quality of life of patients, and even lead to disability. Individuals with cognitive impairment may be unable to perform activities of daily living independently and require care and support from others, increasing the burden on family and society. Therefore, exploring the risk factors of cognitive dysfunction is very important for early intervention measures to avoid its occurrence, to help reduce the severity of cognitive dysfunction and improve the quality of life and social adaptability of patients.

First, our study found that total sleep duration plays an important role in maintaining the normal cognitive function, which is consistent with previous studies. In a study that included 2,472 healthy elderly people and 505 people with mild cognitive impairment, Yuan et al. found that the risk of cognitive impairment was gradually reduced for each additional hour of sleep in elderly people who slept less than 7 h (Yuan et al., 2023). Sleep deprivation is associated with cognitive decline, memory loss, and poor concentration, and the mechanism involves that nighttime sleep is an important time for memory consolidation and neuronal repair, and affects the balance of multiple neurotransmitters in the brain (Girardeau and Lopes-Dos-Santos, 2021). Disruptions of cholinergic and monoaminergic systems have been demonstrated in sleep disorder related cognitive dysfunction (Van Erum et al., 2019). In addition, the cerebrospinal fluid circulation and the glymphatic system play an important role in removing metabolic waste (e.g., amyloid-beta, orexin, tau proteins), which are prone to cognitive dysfunction (Chong et al., 2022). Sleep at night has been confirmed to improve the circulation of cerebrospinal fluid. Therefore, insufficient sleep time can induce the accumulation of metabolic waste by affecting the circulation pathway, which leads to cognitive dysfunction (Lewis, 2021).

Second, we found that participants with sleep latencies of 16–30 min were less likely to experience cognitive dysfunction than those with sleep latencies of less than 16 min. There is no consensus on the effect of sleep latency on cognitive function. In a meat-analysis, Wei et al. found that the sleep latency was prolonged by 6.97 min in older adults with cognitive impairment compared to those with normal cognitive function (Wei et al., 2024). In addition, a previous study using polysomnography to record the sleep structure of Alzheimer’s patients also found that the sleep latency is significantly longer compared to the control group (D'Atri et al., 2021). Sleep latency, as a period of time after going to bed before falling asleep, can also reflect sleep efficiency. However, most current studies support that longer sleep latency is more detrimental to brain health and body homeostasis (Zhong et al., 2022; Akerstedt et al., 2023; Martinez et al., 2023). Our study found that a sleep latency of 16–30 min is better for maintaining cognitive function than a sleep latency of less than 15 min. However, sleep latency within 30 min is considered normal, and our results are from a single center, which may be biased, so it is still worthy of further study.

Third, our study found that participants who were awake more during the night were more likely to have cognitive dysfunction. The more times they were awake at night, the more serious sleep fragmentation existed. Sleep fragmentation has been demonstrated as one of the important causes of neuroinflammation, mainly manifested by increased release of inflammatory factors and activation of microglia in hippocampus (Puech et al., 2023). Besides, impaired vascular endothelial function and disruption of the blood–brain barrier were also observed in sleep fragmentation (Carreras et al., 2014). Therefore, sleep fragmentation could trigger neuroinflammation, especially in hippocampal, compromising the hippocampal-dependent memory consolidation.

Fourth, participants who stayed up more often were more likely to develop cognitive dysfunction. Frequent stay up late indicates the existence of sleep deprivation. The mechanism of cognitive dysfunction caused by sleep deprivation has been formally studied in the following aspects: sleep deprivation can lead to the imbalance of neurotransmitters in the brain, especially neurotransmitters related to learning and memory, such as acetylcholine, which affects the normal operation of cognitive function (Saygin et al., 2017). Long-term sleep deprivation may lead to neuronal damage and synaptic remodeling disorder, affecting the formation of neural networks, information transmission and consolidation of memory, thereby affecting cognitive function (Sharma et al., 2023).

Fifth, having the history of taking sleep medications could trigger the development of cognitive dysfunction. The presence of a history of taking sleep medication was associated with a history of severe sleep disorder, which provides another evidence that poor sleep quality has a negative impact on cognitive function.

In the stratified analysis of our study, we found that age, BMI, and smoking history were associated with cognitive impairment. In terms of age factors, there is a close relationship between age and cognitive dysfunction. With the increase of age, people’s cognitive functions such as attention, memory, learning ability and speed of processing information gradually decline. This cognitive decline can lead to mild cognitive impairment and even progression to dementia such as Alzheimer’s disease. In addition, there are structural and functional changes in the brain, including reduction of gray and white matter, reduction of synaptic connections, and neuronal damage (Oschwald et al., 2019). These changes may affect information processing and transmission in the brain. With aging, the level of inflammation and oxidative stress may increase, leading to neuronal damage and inflammatory response (Luo et al., 2020). Neuroinflammation has been confirmed to be an important mechanism causing cognitive dysfunction.

The present study found that patients with low BMI were more likely to develop cognitive dysfunction. However, there is no agreement on the effect of BMI on cognition. Low BMI may be due to malnutrition. Malnutrition may lead to dystrophic encephalopathy, affecting brain development and function, and thus cognitive function (Corish and Bardon, 2019). Another part of the results showed that there was a certain association between high BMI (obesity) and cognitive impairment. Obesity can lead to problems such as cardiovascular disease, diabetes, and inflammation, which are all associated with cognitive decline and increased risk of dementia (Dye et al., 2017).

As for the effect of smoking on cognition, this study considered nonsmoking as a risk factor for cognitive dysfunction. Although current research also suggests that nicotine may have a certain improvement effect on cognitive function, this does not mean that smoking itself is beneficial to cognitive function. Nicotine is a neurostimulator that may improve cognitive functions such as attention and memory in the short term, but long-term smoking can cause many harmful substances to damage the brain, which in turn affects cognitive function (Valentine and Sofuoglu, 2018; Zhang et al., 2022). Therefore, the relationship between them is still worthy of further exploration.

Despite some important discoveries, this study also had some limitations. The assessment of sleep in this study is based on subjective questionnaire, so the accuracy is different from that of objective monitoring. In addition, the results of this study were analyzed in a single center, so the extrapolation of the results is limited. Notwithstanding its limitations, this study provided important clinical meaning. In our study, we found that poor sleep quality was associated with cognitive impairment. Therefore, community physicians should pay more attention to sleep problems to reduce the incidence of cognitive dysfunction.

## Conclusion

Poor sleep quality may be associated with cognitive decline. These findings provide important clues for further research on the relationship between sleep and cognitive function, and highlight the importance of sleep for brain health. More long-term, longitudinal studies would be helpful to further validate these associations and provide more specific guidance and interventions for the prevention of cognitive dysfunction.

## Data Availability

The original contributions presented in the study are included in the article/Supplementary material, further inquiries can be directed to the corresponding author.
