# Supplementary material for: Association of sleep quality with cognitive dysfunction in middle-aged and elderly adults: a cross-sectional study in China
Source: Front Aging Neurosci. 2024 Sep 16;16:1417349. doi: 10.3389/fnagi.2024.1417349 (PMC11439658; doi:10.3389/fnagi.2024.1417349)
Supplement: Supplementary file 1 [file Table_1.DOCX]

Supplementary Material

Table S1. Stratified analysis: Risk of cognitive dysfunction based on the total sleep duration.

|  | Cognitive dysfunction | | | | | | |
| --- | --- | --- | --- | --- | --- | --- | --- |
|  | Total sleep duration (6-7.9h) | | |  | Total sleep duration (>8h) | | |
|  | OR (95% CI) | *P* | *P* interaction |  | OR (95% CI) | *P* | *P* interaction |
| Age (years) |  |  | **0.042** |  |  |  | 0.471 |
| 40-59 | 0.44 (0.25-0.78) | 0.005 |  |  | 0.45 (0.19-1.04) | 0.061 |  |
| 60-69 | 0.47 (0.29-0.76) | 0.002 |  |  | 0.48 (0.20-1.15) | 0.101 |  |
| ≥70 | 1.63 (0.58-4.57) | 0.356 |  |  | 1.15 (0.22-5.92) | 0.865 |  |
| Gender |  |  | 0.075 |  |  |  | 0.424 |
| male | 0.41 (0.25-0.67) | 0.002 |  |  | 0.42 (0.20-0.90) | <0.001 |  |
| female | 0.71 (0.44-1.15) | 0.168 |  |  | 0.71 (0.31-1.61) | 0.405 |  |
| BMI (kg/m^2^) |  |  | 0.076 |  |  |  | **0.043** |
| ＜24 | 0.84 (0.45-1.57) | 0.590 |  |  | 1.16 (0.47-2.88) | 0.753 |  |
| ≥24 | 0.46 (0.30-0.70) | <0.001 |  |  | 0.39 (0.19-0.79) | 0.010 |  |
| Hypertension (n, %) |  |  | 0.863 |  |  |  | 0.851 |
| yes | 0.54 (0.34-0.87) | 0.011 |  |  | 0.60 (0.28-1.29) | 0.192 |  |
| no | 0.61 (0.37-0.99) | 0.049 |  |  | 0.57 (0.25-1.26) | 0.162 |  |
| Diabetes (n, %) |  |  | 0.950 |  |  |  | 0.921 |
| yes | 0.60 (0.26-1.40) | 0.236 |  |  | 0.54 (0.15-1.99) | 0.351 |  |
| no | 0.57 (0.39-0.83) | 0.004 |  |  | 0.58 (0.31-1.07) | 0.083 |  |
| Hyperlipidemia (n, %) |  |  | 0.786 |  |  |  | 0.242 |
| yes | 0.60 (0.39-0.92) | 0.020 |  |  | 0.45 (0.21-0.95) | 0.037 |  |
| no | 0.51 (0.29-0.90) | 0.020 |  |  | 0.76 (0.33-1.77) | 0.523 |  |
| Smoking (n, %) |  |  | 0.152 |  |  |  | 0.609 |
| yes | 0.33 (0.15-0.73) | 0.006 |  |  | 0.37 (0.11-1.27) | 0.112 |  |
| no | 0.62 (0.42-0.91) | 0.014 |  |  | 0.62 (0.33-1.17) | 0.139 |  |
| Drinking |  |  | 0.111 |  |  |  | 0.121 |
| Yes | 0.32 (0.14-0.71) | 0.005 |  |  | 0.12 (0.02-0.70) | 0.019 |  |
| No | 0.64 (0.44-0.94) | 0.021 |  |  | 0.73 (0.40-1.31) | 0.291 |  |

BMI: Body mass index

Table S2. Stratified analysis: Risk of cognitive dysfunction based on the sleep latency.

|  | Cognitive dysfunction | | | | | | | | | | |
| --- | --- | --- | --- | --- | --- | --- | --- | --- | --- | --- | --- |
|  | Sleep latency (16-30 min) | | |  | Sleep latency (30-60 min) | | |  | Sleep latency (>1 h) | | |
|  | OR (95% CI) | *P* | *P* interaction |  | OR (95% CI) | *P* | *P* interaction |  | OR (95% CI) | *P* | *P* interaction |
| Age (years) |  |  | 0.278 |  |  |  | 0.693 |  |  |  | **0.031** |
| 40-59 | 0.25 (0.15-0.43) | <0.001 |  |  | 0.36（0.11-1.21） | 0.099 |  |  | 4.02（1.17-13.84） | 0.028 |  |
| 60-69 | 0.48 (0.28-0.85） | 0.011 |  |  | 0.53（0.17-1.61） | 0.263 |  |  | 1.40（0.48-4.11） | 0.541 |  |
| ≥70 | 0.23 (0.07-0.76） | 0.016 |  |  | 0.90（0.18-4.41） | 0.897 |  |  | 0.45（0.08-2.40） | 0.384 |  |
| Gender |  |  | 0.530 |  |  |  | 0.342 |  |  |  | 0.666 |
| male | 0.37 (0.23-0.62) | <0.001 |  |  | 0.80 (0.29-2.24) | 0.677 |  |  | 1.865(0.41-6.72) | 0.482 |  |
| female | 0.29 (0.17-0.59) | <0.001 |  |  | 0.37(0.15-1.01) | 0.053 |  |  | 1.29 (0.54-3.12) | 0.568 |  |
| BMI (kg/m^2^) | |  | 0.779 |  |  |  | 0.483 |  |  |  | 0.756 |
| ＜24 | 0.30 (0.16-0.55） | <0.001 |  |  | 0.32（0.07-1.40） | 0.140 |  |  | 1.11（0.33-3.73） | 0.868 |  |
| ≥24 | 0.34 (0.22-0.53） | <0.001 |  |  | 0.66（0.30-1.48） | 0.313 |  |  | 1.37（0.52-3.61） | 0.525 |  |
| Hypertension (n, %) | |  | 0.559 |  |  |  | 0.802 |  |  |  | 0.123 |
| yes | 0.36 (0.22-0.61) | <0.001 |  |  | 0.49 (0.18-1.33） | 0.164 |  |  | 2.22（0.85-5.82） | 0.106 |  |
| no | 0.29 (0.17-0.49） | <0.001 |  |  | 0.56 (0.21-1.47） | 0.238 |  |  | 0.60（0.16-2.18） | 0.434 |  |
| Diabetes (n, %) | |  | 0.536 |  |  |  | 0.831 |  |  |  | 0.482 |
| yes | 0.37 (0.15-0.89) | 0.027 |  |  | 0.54 (0.06-4.81) | 0.584 |  |  | 0.80 (0.09-7.55) | 0.847 |  |
| no | 0.31 (0.21-0.47) | <0.001 |  |  | 0.51 (0.24-1.07) | 0.076 |  |  | 1.40 (0.63-3.10) | 0.414 |  |
| Hyperlipidemia (n, %) | |  | 0.742 |  |  |  | 0.530 |  |  |  | 0.740 |
| yes | 0.34 (0.22-0.55) | <0.001 |  |  | 0.47 (0.19-1.13) | 0.091 |  |  | 1.30 (0.51-3.32) | 0.580 |  |
| no | 0.31 (0.17-0.56) | <0.001 |  |  | 0.71 (0.23-2.21) | 0.553 |  |  | 1.36 (0.39-4.69) | 0.627 |  |
| Smoking (n, %) | |  | 0.067 |  |  |  | 0.649 |  |  |  | 0.164 |
| yes | 0.14 (0.05-0.37) | <0.001 |  |  | 0.40 (0.08-2.00) | 0.266 |  |  | 6.51 (0.66-64.12) | 0.108 |  |
| no | 0.38 (0.26-0.57) | <0.001 |  |  | 0.57 (0.26-1.23) | 0.153 |  |  | 1.13 (0.50-2.53) | 0.771 |  |
| Drinking |  |  | 0.208 |  |  |  | 0.748 |  |  |  | 0.575 |
| Yes | 0.16 (0.05-0.49) | 0.001 |  |  | 0.57 (0.11-2.94) | 0.505 |  |  | 2.38 (0.16-36.44) | 0.534 |  |
| No | 0.36 (0.25-0.54) | <0.001 |  |  | 0.52 (0.24-1.13) | 0.097 |  |  | 1.26 (0.58-2.72) | 0.562 |  |

BMI: Body mass index

Table S3. Stratified analysis: Risk of cognitive dysfunction based on the number of awakenings.

|  | Cognitive dysfunction | | | | | | | | | | | |
| --- | --- | --- | --- | --- | --- | --- | --- | --- | --- | --- | --- | --- |
|  | Number of awakenings (1) | | |  | Number of awakenings (2) | | |  | | Number of awakenings (≥3) | | |
|  | OR (95% CI) | *P* | *P* interaction |  | OR (95% CI) | *P* | *P* interaction |  | OR (95% CI) | | *P* | *P* interaction |
| Age (years) |  |  | **<0.001** |  |  |  | 0.858 |  |  | |  | 0.130 |
| 40-59 | 3.14 (1.84-5.35) | <0.001 |  |  | 1.70 (0.61-4.73) | 0.306 |  |  | 4.01 (1.23-13.06) | | 0.021 |  |
| 60-69 | 0.72 (0.44-1.17) | 0.186 |  |  | 1.34 (0.66-2.71) | 0.415 |  |  | 1.32 (0.56-3.12) | | 0.521 |  |
| ≥70 | 4.14 (1.33-12.88) | 0.014 |  |  | 1.67 (0.41-6.79) | 0.473 |  |  | 4.33 (0.80-23.48) | | 0.089 |  |
| Gender |  |  | 0.516 |  |  |  | 0.520 |  |  | |  | 0.570 |
| Male | 1.49 (0.95-2.35) | 0.086 |  |  | 1.74 (0.86-3.50) | 0.123 |  |  | 2.63 (1.07-6.50) | | 0.036 |  |
| female | 1.82 (1.13-2.93) | 0.014 |  |  | 1.63 (0.75-3.52) | 0.214 |  |  | 2.18 (0.88-5.43) | | 0.093 |  |
| BMI (kg/m^2^) | |  | 0.680 |  |  |  | 0.680 |  |  | |  | 0.566 |
| ＜24 | 1.92 (1.06-3.48) | 0.032 |  |  | 1.43 (0.55-3.74) | 0.468 |  |  | 2.04 (0.67-6.23) | | 0.211 |  |
| ≥24 | 1.53 (1.02-2.27) | 0.038 |  |  | 1.68 (0.91-3.13) | 0.099 |  |  | 2.58 (1.19-5.58) | | 0.017 |  |
| Hypertension (n, %) | |  | 0.266 |  |  |  | 0.648 |  |  | |  | 0.302 |
| yes | 2.06 (1.25-3.39) | 0.005 |  |  | 2.08 (1.00-4.31) | 0.049 |  |  | 1.83 (0.75-4.50) | | 0.185 |  |
| no | 1.40 (0.90-2.18) | 0.137 |  |  | 1.33 (0.63-2.79) | 0.457 |  |  | 3.17 (1.29-7.79) | | 0.012 |  |
| Diabetes (n, %) | |  | 0.501 |  |  |  | 0.581 |  |  | |  | 0.061 |
| yes | 1.34 (0.61-2.94) | 0.473 |  |  | 0.84 (0.21-3.41) | 0.806 |  |  | 0.87 (0.17-4.53) | | 0.872 |  |
| no | 1.73 (1.20-2.49) | 0.003 |  |  | 1.78 (1.01-3.12） | 0.047 |  |  | 3.13 (1.57-6.26) | | 0.001 |  |
| Hyperlipidemia (n, %) | |  | 0.890 |  |  |  | 0.256 |  |  | |  | 0.917 |
| yes | 1.70 (1.12-2.57) | 0.013 |  |  | 1.32 (0.66-2.62) | 0.436 |  |  | 2.15 (0.93-4.94) | | 0.072 |  |
| no | 1.53 (0.89-2.63) | 0.126 |  |  | 2.00 (0.89-4.49) | 0.095 |  |  | 3.17 (1.17-8.54) | | 0.023 |  |
| Smoking (n, %) | |  | **0.005** |  |  |  | 0.519 |  |  | |  | 0.617 |
| yes | 0.78 (0.40-1.53) | 0.469 |  |  | 1.04 (0.33-3.33) | 0.947 |  |  | 1.94 (0.31-11.98) | | 0.478 |  |
| no | 2.19 (1.48-3.25) | <0.001 |  |  | 2.03 (1.13-3.64) | 0.018 |  |  | 2.82 (1.42-5.58) | | 0.003 |  |
| Drinking |  |  | 0.777 |  |  |  | 0.776 |  |  | |  | 0.469 |
| Yes | 1.56 (0.73-3.32) | 0.252 |  |  | 1.32 (0.37-4.68) | 0.666 |  |  | 2.98 (0.52-17.12) | | 0.221 |  |
| No | 1.71 (1.19-2.47) | 0.004 |  |  | 1.70 (0.96-3.02) | 0.067 |  |  | 2.22 (1.12-4.38) | | 0.022 |  |

BMI: Body mass index
